# Anterolateral and Medial Locking Compression Plates for the Management of Distal Tibial Fractures: A Comparative Prospective Study

**DOI:** 10.7759/cureus.44235

**Published:** 2023-08-28

**Authors:** Dinesh Kumar, Ankit Mittal, Jasveer Singh, Harish Kumar, Prashant P Singh, Akash Kumar, Ashish Singhania, Ravi Kant

**Affiliations:** 1 Department of Orthopaedics, Uttar Pradesh University of Medical Sciences, Etawah, IND

**Keywords:** distal tibia locking compression plate, distal tibia fractures, internal fixation, close reduction, open reduction

## Abstract

Introduction: Open/close reduction (OR/CR) and internal fixation (IF) of displaced fractures of distal tibia with either a medial or anterolateral plate is a commonly performed procedure. Anterolateral plating avoids an incision along the medial subcutaneous border of tibia and has been shown to have reduced risk of wound complications. The aim of our study was to determine the functional outcome of these fractures treated with anterolateral and medial distal tibial locking compression plates.

Methods: This was a prospective study that included 60 patients with distal tibial fractures (close or grade I open injury) divided into two groups with 30 patients in each where one group was treated with OR/CR and IF using an anterolateral distal tibial locking plate (Group A) and the other using a medial distal tibial locking plate (Group B). The duration of surgery and intraoperative blood loss and time to union were recorded for all the patients. Functional evaluation was done at one year in terms of pain, function and alignment using the American Orthopaedic Foot and Ankle Society (AOFAS) ankle-hindfoot scale, and complications, if any, were noted.

Results: Both the groups were comparable in terms of age, gender, time of presentation, AO classification and presence of wound. The mean duration of surgery as well as the intraoperative blood loss were more in the anterolateral plate group than in the medial plate group, but the difference was statistically insignificant. Ten patients (33%) with medial plates had symptomatic hardware and 7 (23.3%) underwent removal while only 3 (10%) patients in the anterolateral plate group had similar complaints and none had to undergo removal. Two patients with anterolateral plate and one with medial plate had malunion. The mean time to fracture union as well as the rate of infection was less and the functional outcome at one year was better in the group treated with anterolateral plates as compared to the one with medial plates, but the difference again was not statistically significant for all the parameters.

Conclusion: With reduced risk of soft tissue complications and by obviating the need for implant removal, anterolateral plates can prove to be a better alternative to the medial plates especially in elderly patients in the management of these fractures.

## Introduction

Tibial pilon (tibial plafond) is a term used to describe the fractures of the distal tibia caused by the talus acting as a hammer and impacting against it [1]. They constitute about 1%-10% of all fractures of the lower limbs [2]. Their treatment is still an enigma owing to the scant vasculature around the ankle. The subcutaneous presence of bone further adds to wound-healing problems. Surgical treatment is challenging due to difficulty in attaining and maintaining good reduction by intramedullary nails and a predisposition to infection and non-union caused by dissection around the fracture site while inserting the plate [3,4]. To achieve anatomical reduction and firm fixation, an open reduction and internal fixation with a plate may be employed. The medial tibial plate placement technique is commonly utilised; however, it increases the risk of infection, wound disruption, and implant-related complications despite providing great exposure [5]. A newer technique, minimally invasive percutaneous medial plating, is technically exacting, and attaining anatomical reduction utilising it is a challenge [6]. Moreover, fixation of fibular fractures demands an additional incision on the lateral side of the shin with this approach. Anterolateral plating, a novel technique, makes use of the thick muscle covering the lateral aspect of the tibia. For lateral side distal tibia fractures with fibula fracture and syndesmotic injury, this method is preferred because the posterior tibial fracture fragments can be observed by mobilising the fibular chaput fragment [7].

Yenna et al. did a biomechanical study and found no statistically significant difference between the stiffness of lateral and medial plates in compression and torsion tests [8]. Studies that directly compare the two plating techniques on human subjects are lacking in the literature. The purpose of the current study was to evaluate the clinical and functional outcomes of distal tibial fractures treated with anterolateral and medial distal tibia locking compression plates.

## Materials and methods

A total of 60 patients with distal tibia fractures, with or without fibula, who met the inclusion and exclusion criteria were enrolled in this randomised, prospective investigation between January 2020 and March 2021, at Uttar Pradesh University of Medical Sciences, Saifai. Purposive sampling method was used for sample collection. Patients underwent block randomization with a random number table to determine whether they would undergo fixation with a medial or anterolateral distal tibial locking plate. The radiological and functional results were evaluated and compared. Our institution's ethical review board approved the proforma of the study. Before enrolling any patients in this trial, we made sure to get their full and informed consent.

Patients aged 18 years or above, with minimum 12 months of follow-up, with open or closed fractures (Gustilo grade 1), and polytrauma without major upper or lower limb fractures on the injured side were included in the study. Exclusion criteria included significant associated injury to the ipsilateral lower extremity, significant comorbidity in the ipsilateral lower extremity, neurovascular complications, inability to walk before the injury, pathological fractures, and long-term use of corticosteroids.

All open fractures were thoroughly irrigated, debrided, and put on intravenous antibiotics immediately after admission. An above knee slab or calcaneal skin traction was applied while the patients waited for surgery. Definitive management was undertaken when the soft tissue condition permitted. In this research, we used the AO/ASIF classification system to classify distal tibial fractures [9]. The final tally of patients, with demographics, in each group is shown in Table 1.

**Table 1:** Demographic data and fracture patterns for both the groups RTA, road traffic accident

		Group A (anterolateral plate, 30 cases)	Group B (medial plate, 30 cases)	p-value
Mean age ± SD (years)		37.4 ± 10.0	41.8 ± 12.9	0.15
Gender (no. of cases)	Male	26	20	0.07
	Female	04	10	
Side (no. of cases)	Right	18	17	0.79
	Left	12	13	
Mode of trauma (no. of cases)	RTA	16	15	
	Slip and fall	09	07	
	Fall from height	05	07	
	Assault	00	01	
Type of fracture (no. of cases)	Close	26	24	0.49
	Open	04	06	
Type of fracture (AO classification; no. of cases)	43-A	08	10	0.58
	43-B	15	11	
	43-C	07	09	
Duration between injury and surgery (days), mean ± SD		10.3 ± 3.3	09.5 ± 2.3	0.30
Associated fibula fracture present (no. of cases)		25	23	0.75

**Figure 1:**
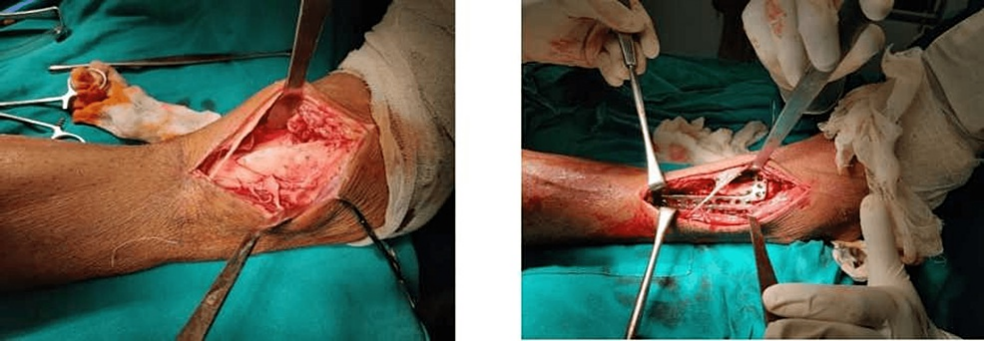
Anterolateral approach for distal tibial fractures

Patients were divided into two groups. Group A, which included 30 patients, had internal fixation and open reduction using an anterolateral plating technique [9]. A skin incision over the distal leg in line with the 4th ray was made, and the superficial peroneal nerve and its arborizations were located and retracted (Figure 1).

**Figure 2:**
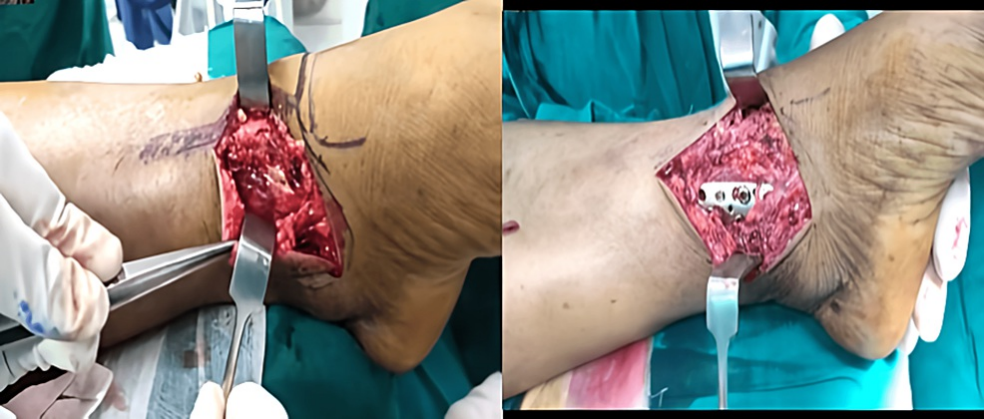
Medial approach for distal tibial fractures

Group B, which also included 30 patients, had internal fixation with a medial tibial locking plate. The plate was slid proximally through a 4- to 5-cm longitudinal incision centred over the medial malleolus, and the fracture, if necessary, was exposed by a second incision immediately lateral to the tibial shin (Figure 2) [10].

In both groups, the fracture fragments were provisionally fixed with K-wire before definitive fixation with the plate. We fixed a minimum of six cortices both proximally and distally with either implant. A locking recon plate or intramedullary nail was used to treat any fibula fracture, if present, after its close or open reduction. Operating time (min) and blood loss (ml) to the nearest multiple of 5 were recorded in all the cases.

Postoperatively, the limb was kept in a below knee plaster of Paris (POP) slab for three weeks. Patients were seen first for suture removal at two weeks, then at four weeks, and finally once a month for a full year. After the appearance of radiographic signs of union, partial weight bearing was allowed, and full weight bearing was allowed only if radiographic evidence of union was detected by the presence of bridging callus and crossing trabeculae in at least three of the four cortices in both the anteroposterior and lateral views. A more than 5 degree deformity in any plane, a 10 degree internal rotation, a 15 degree external rotation, or a 2 cm shortening was considered an example of malunion. We defined delayed union as union after the six-month mark and non-union as persistent failure to unite despite further surgical intervention.

After a year of monitoring, functional status was assessed using the American Orthopaedic Foot and Ankle Society (AOFAS) ankle-hindfoot scale, which is a 100-point scale that includes pain (40 points), function (50 points), and alignment (10 points) assessments [11]. The results were categorized as excellent, good, fair, and poor.

We used the t-test to analyse the difference in means for different parameters. Two-tailed p-values were derived from the test, and 95% confidence intervals were established around the sensitivity percentage using the normal approximation approach. Analyses of paired categorical variables were conducted using Fischer's and chi-square tests. The statistical analyses were done using SPSS, version 23 (IBM Corp., Armonk, NY). Statistical significance was defined as a p-value less than 0.05.

## Results

The final evaluation was done for patients in each group. Comparable mean ages were shared by patients in Groups A and B (37.4 ± 10.0 and 41.8 ± 12.9). Sex distribution, mechanism of injury, concomitant wound or fibula fracture, time between injury and surgery, and AO fracture type were all comparable across the two groups as well. The duration of surgery and mean blood loss were both higher in the anterolateral plating group as compared to the medial plating group, but these differences did not achieve statistical significance (Table 2).

**Table 2:** Intraoperative results for both the groups

	Group A (anterolateral plate)	Group B (medial plate)	p-value
Duration of surgery (min), mean ± SD	80.3 ± 29.1	77.9 ± 26.7	0.74
Blood loss (ml), mean ± SD	172.8 ± 18.2	167.8 ± 22.9	0.35

All incidences of superficial infection (three in the group with anterolateral plating and seven in the group with medial plating) were detected prior to suture removal and managed nonoperatively with routine dressings and intravenous antibiotics. There were four cases of wound dehiscence: one in the anterolateral plate group and three in the medial plate group, and they all presented before suture removal. Two out of them, one in either group, required thorough surgical debridement, while the other two were managed conservatively with dressings and intravenous antibiotics (Table 3).

**Table 3:** Results and complications in both the groups

	Group A (anterolateral plate)	Group B (medial plate)	p-value
Mean time to union (weeks)	20.27 ± 05.4	20.8 ± 06.4	0.73
Malunion (no. of cases)	02	01	0.55
Superficial infection (no. of cases)	03	07	0.17
Wound dehiscence (no. of cases)	01	03	0.30
Symptomatic hardware (no. of cases)	03	10	0.03
Hardware removal (no. of cases)	00	07	0.005
Ankle-hindfoot scale score (100 points)	78.7 ± 10.5	76.8 ± 08.7	0.45

The incidence of hardware-related issues was higher in the medial plate group than in the anterolateral plate group. Ten patients in the medial plating group complained of prominent hardware, and seven out of them underwent plate removal on their own will, while only three patients in the anterolateral plating group had this complaint and none of them requested plate removal (Table 3). Delay in union was an issue that affected five patients who had anterolateral plating and eight patients who received medial plating. Two patients in the anterolateral plating group and one patient in the medial plating group had malunion. We did not come across any cases of non-union in our study.

**Figure 3:**
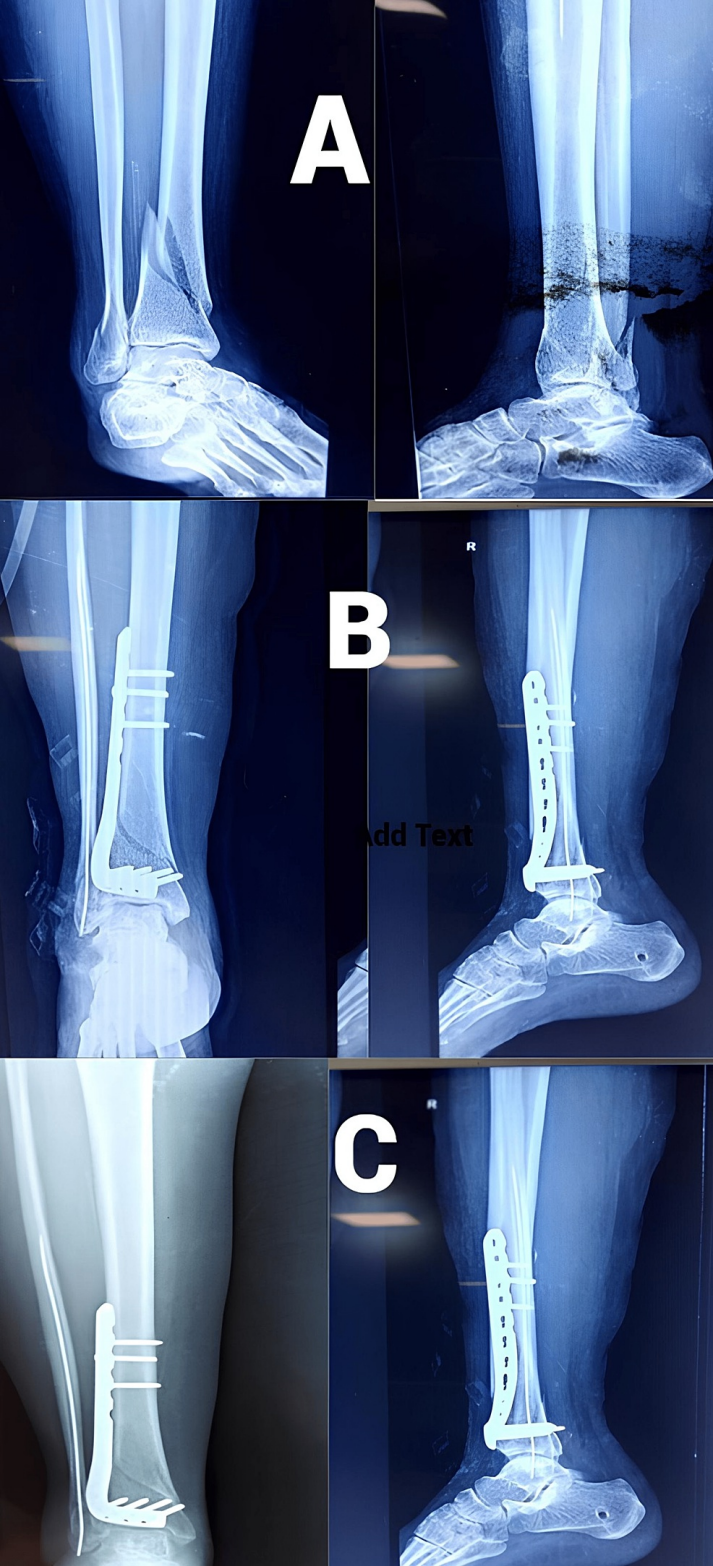
Case 1 with anterolateral plating (Group A) (A) Pre-operative X-ray showing fracture of the distal tibia and fibula, (B) immediate post-operative X-ray, (C) post-operative X-ray at the one-year follow-up

**Figure 4:**
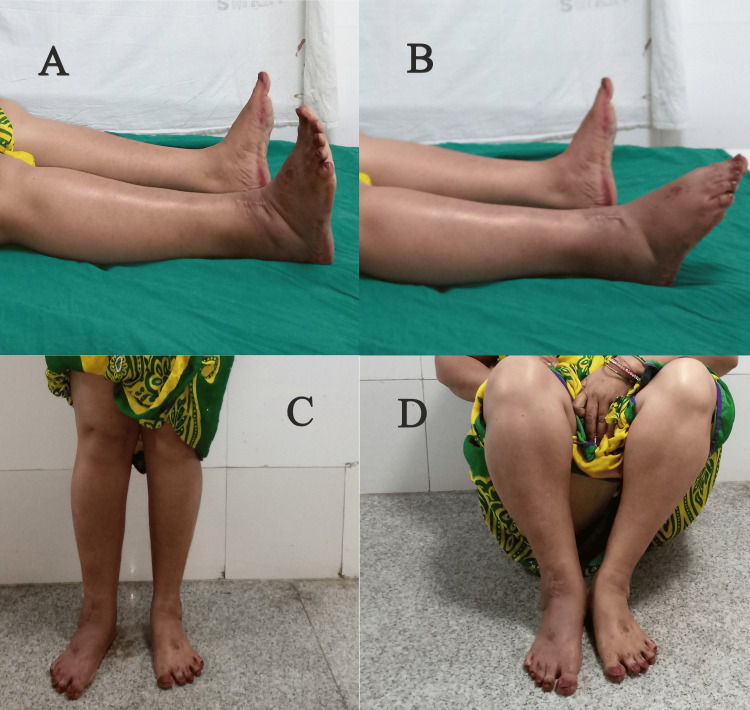
Clinical outcomes in Case 1 at one year (A) Ankle dorsiflexion, (B) ankle plantarflexion, (C) standing, (D) squatting

**Figure 5:**
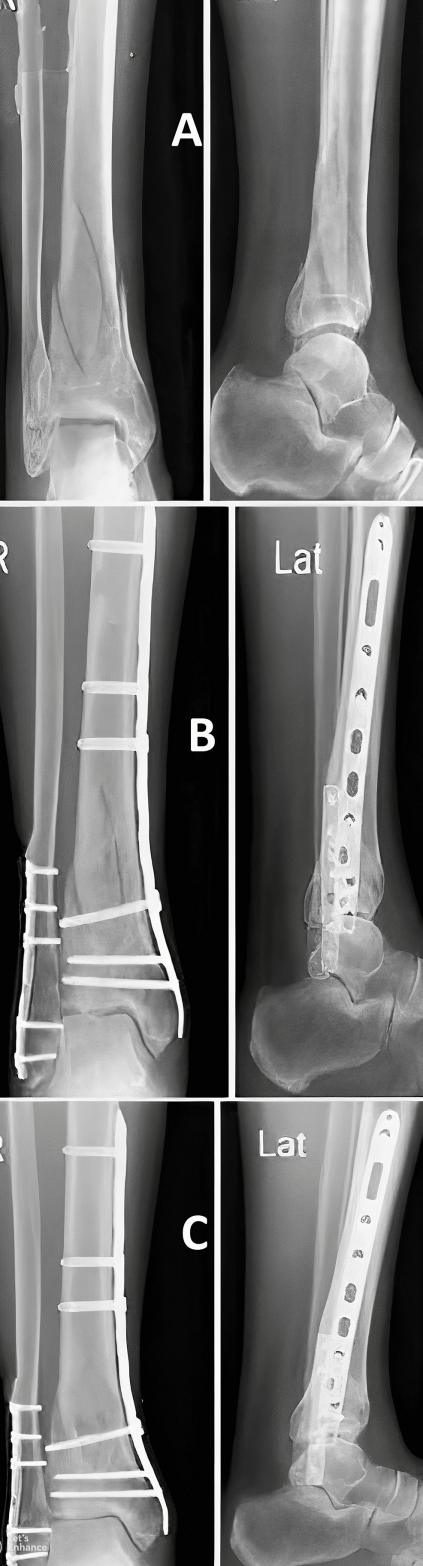
Case 2 with medial plating (Group B) (A) Pre-operative X-ray showing fracture of the distal tibia and fibula, (B) immediate post-operative X-ray, (C) post-operative X-ray at one-year follow-up

**Figure 6:**
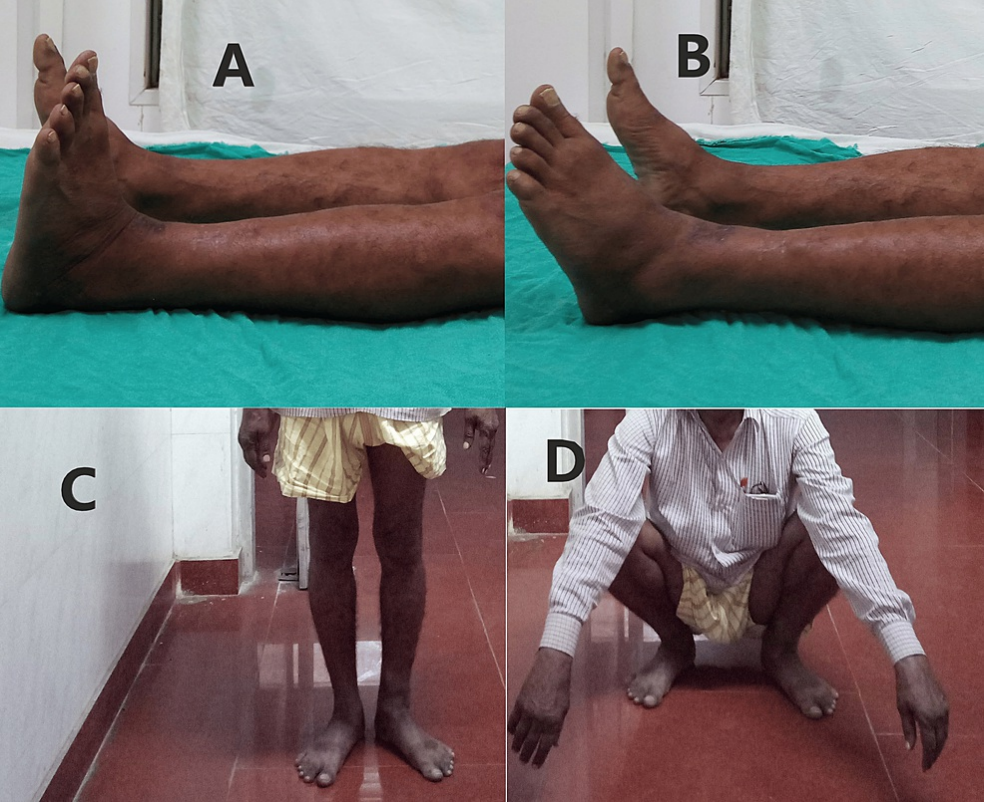
Clinical outcomes in Case 2 at one year (A) Ankle dorsiflexion, (B) ankle plantarflexion, (C) standing, (D) squatting

**Figure 7:**
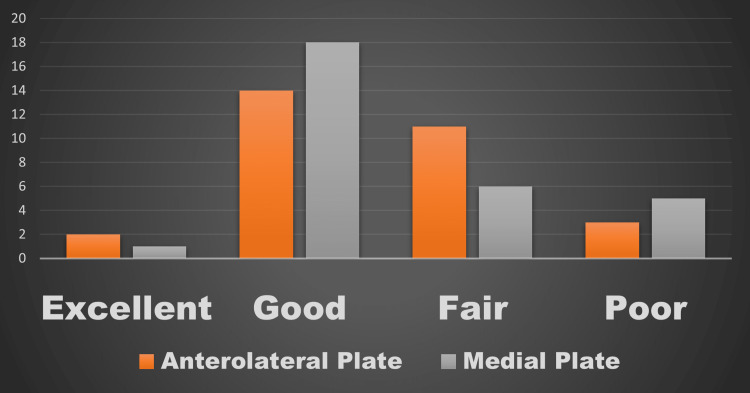
Functional results (based on AOFAS scores) in both the groups with anterolateral and medial plating techniques for distal tibial fractures AOFAS, American Orthopaedic Foot and Ankle Society

Functional results at the one-year follow-up were evaluated using the AOFAS score. The anterolateral plating group fared better in terms of this score, but the difference was not statistically significant (Figures 3-6). The percentage of cases in each category that received an "excellent," "good," "fair," or "poor" evaluation is shown in Figure 7.

## Discussion

Although conservative treatment options for distal tibial fractures have been tried, they have been linked to an increased risk of malunion and limb shortening [11]. Intramedullary nails do not fit into the cavity snugly and often result in malunion, and moreover, they cannot be used in fractures extending into the joint [12,13].

Anterolateral or medial application of a locking plate to the distal tibia is an option for treating these fractures, and both approaches have their advantages and disadvantages. The conventional medial tibial locking plating technique provides acceptable reduction but is notorious to cause soft tissue problems [14,15]. Anterolateral plating avoids or at least reduces these issues by capitalising on the larger soft tissue envelope provided by the anterior compartment muscles on the lateral aspect of the bone. Lee et al., in their comparative study of these two approaches, described a good functional outcome with both but fewer complications like soft tissue infection with lateral plating [16,17]. We also reported lesser incidence of superficial infection, wound dehiscence, prominent hardware and need for hardware removal with anterolateral plating than with medial plating.

We observed more operating time and blood loss with lateral than with medial plating, but the difference was not significant statistically. This may be attributed to the need for a longer skin incision and subsequently longer dissection and closure time with the anterolateral than with the medial approach. Manninen et al., in 20 patients, used the anterolateral approach and discovered this procedure to be technically demanding [18]. Garg et al., in their comparative study between lateral and medial plates with 18 patients in either group, reported significantly less operating time with lateral plates than with medial plates and did not experience any difficulties with the lateral approach [19]. They also reported less soft tissue complications, prominent hardware and need for hardware removal with lateral plating than with medial plating as was the case in our study.

All the cases in both the groups evidenced union of their fracture at or before eight months (Figures 3, 5). Five cases with anterolateral plates and eight with medial plates had their fracture union after six months. We saw two cases of malunion with the anterolateral plate and one with the medial plate. In their retrospective study of 45 patients treated with both approaches, Piątkowski et al. showed that non-union occurred in 26.6% of patients and shin axis abnormalities occurred in 13.3% of patients [20]. Bony union complications were more with the anterior approach (50%) than with the medial approach (16.6%) in their study. Advancement in the locking plating system might have led to the reduction in these complications in the recent years.

With regard to the functional aspect, both the groups performed equally well without any significant difference at the one-year follow-up when evaluated using the AOFAS scores (Figures 4, 6). Garg et al. showed that after at least five months of follow-up, functional results according to the Tenny and Wiss criteria and ankle joint range of motion were almost equal across the two groups [19]. Encinas-Ullán et al., using the AOFAS score in their prospective study of 40 patients, failed to find any meaningful variations in radiographic and clinical outcomes after two years of follow-up [21].

## Conclusions

To conclude, the type of plate and the surgical approach may not have much influence on the final outcome, but the anterolateral plate can be preferred in patients where the surgeon anticipates healing problems, like with notable soft tissue injury or thinner soft tissue envelope around the distal tibia. Anterolateral plating can also help in the reduction of implant-related complaints like hardware prominence and requests for its removal. Since our research was conducted on a small sample of patients and in a very short time frame, it cannot provide insights into the long-term outcomes and consequences of any of these procedures. More trials that are appropriately designed and powered are required.
